# New advances in Traditional Chinese Medicine interventions for epilepsy: where are we and what do we know?

**DOI:** 10.1186/s13020-025-01088-z

**Published:** 2025-03-18

**Authors:** Minjuan Sun, Xiaoyun Qiu, Zhijian Yuan, Cenglin Xu, Zhong Chen

**Affiliations:** Key Laboratory of Neuropharmacology and Translational Medicine of Zhejiang Province, Huzhou Central Hospital, the Fifth School of Clinical Medicine of Zhejiang Chinese Medical University, College of Pharmaceutical Science, Zhejiang Chinese Medical University, Hangzhou, Zhejiang China

**Keywords:** Epilepsy, Traditional Chinese Medicine, Herbal medicine, Prescription, Acupuncture, Epileptic comorbidities

## Abstract

Epilepsy, one of the most common neurological diseases, affects more than 70 million people worldwide. Anti-seizure drugs targeting membrane ion channels or GABAergic neurotransmission are the first choices for controlling seizures, whereas the high incidence of pharmacoresistance and adverse effects largely restrict the availability of current anti-seizure drugs (ASDs). Traditional Chinese Medicine (TCM) has shown historical evidence-based therapeutic effects for neurological diseases including epilepsy. But until the late 1990s, great efforts in both clinical and experimental fields advanced TCM interventions for epilepsy from evidence-based practices to more systematic neuropharmacological significance, and show new lights on preferable management of epilepsy in the last decade. This review summarized the advances of applying TCM interventions (ranging from herbal medicines and their active ingredients to other strategies such as acupuncture) for epilepsy, followed by associated mechanism theories. The therapeutic potential of TCM interventions for epilepsy as well as its comorbidities turns from somehow debatable to hopeful. Finally, some prospects and directions were proposed to drive further clinical translational research. The future directions of TCM should aim at not only deriving specific anti-epileptic molecules but also illustrating more precise mechanisms with the assistance of advanced multifaceted experimental tools.

## Introduction

Epilepsy, one of the most prevalent and severe neurological diseases with a nearly ~ 1% prevalence worldwide, is notorious for its life-threatening unprovoked recurrent seizure [1, 2]. In China, approximately 10 million people are diagnosed with epilepsy, and the annual management costs of epilepsy emerge as great burdens for both patients with epilepsy (PWEs) and the society [3]. Anti-seizure drugs (ASDs) primarily target membrane ion channels or neural transmission to control seizures [4]. There are notable limitations in the current treatment of ASDs. Long-term, or life-long medications would cause unwanted intolerable side effects including drowsiness, ataxia, cognitive decline et al. [5, 6]. And in elderly patients, ASDs can interfere with the metabolism of other cardiovascular and antihypertensive medications [7]. In infants, blood drug concentrations differ from those observed in adult patients [8]. In pregnant women, the distribution of ASDs within the body changes, posing a risk of teratogenesis [9]. Additionally, during lactation, these drugs may be secreted into breast milk [10]. More importantly, one-third of the PWEs remain relapse after taking more than two ASDs, and develop into pharmacoresistant epilepsy [11, 12]. Also, this unsatisfactory situation of current ASDs pushes forward an urgent need for searching alternative interventions for epilepsy.

Treating epilepsy in China has a long history. The *Yellow Emperor*’*s Internal Classic*, compiled between 770–221 B.C., first defined epilepsy as a congenital disease with fetal origins. Detailed descriptions of epileptic symptoms and etiologies have also been summarized by ancient Chinese physicians [13]. Along with the cognization of the disease, Traditional Chinese Medicine (TCM) provided interventions in an evidence-based manner for epilepsy. However, ancient records of effective TCM interventions for epilepsy are mainly restricted to a single case or small samples [14]. The lack of large-sample efficacy confirmation and in-depth mechanism studies raises doubts about TCM's effectiveness for epilepsy. Fortunately, the development of various animal models and their translational significance for testing new anti-epileptic drugs have provided essential foundations for advancing TCM interventions for epilepsy since the twenty-first century [15]. The advances achieved in laboratory studies over the last decade have further facilitated the clinical translation of novel medicine or treatments derived from TCM.

In this review, we summarized the recent advances in TCM interventions for epilepsy, elucidate their potential mechanisms, and proposes future directions to promote integrated traditional Chinese and modern medicine for better epilepsy management.

## Understanding epilepsy in traditional Chinese medicine: think in a Chinese way

**Fig. 1:**
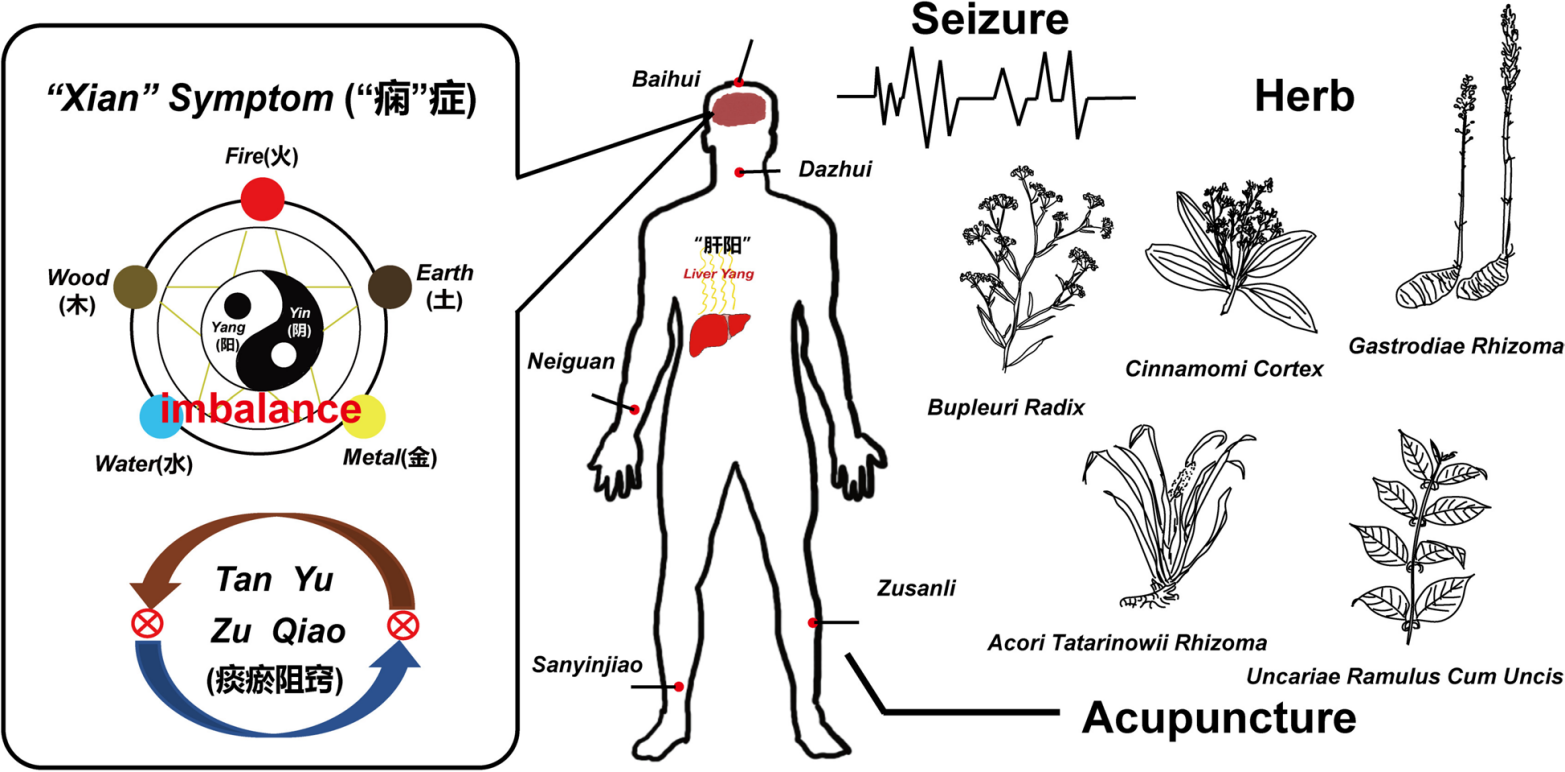
The pathogenesis and treatment of epilepsy under the theory of Traditional Chinese Medicine. Left part: The imbalance of *Yin Yang* or *Wu Xing* in the internal body, and the rise of *Liver Yang* can contribute to *Tan Yu Zu Qiao*, thus resulting in the occurrence of seizures according to TCM theory. Right part: The main TCM treatments for epilepsy including herbal medicine and acupuncture. Herbal medicines with anti-epileptic effects include *Bupleuri Radix*, *Cinnamomi Cortex*, *Acori Tatarinowii Rhizoma*, *Gastrodiae Rhizoma*, *Uncariae Ramulus Cum Uncis* et al. Middle part: Acupuncture points that can produce anti-epileptic effects include *Baihui*, *Dazhui*, *Neiguan*, *Sanyinjiao*, *Zusanli* et al.

Distinct from modern medicine, TCM has its own theoretical frameworks for comprehending diseases with the representative “*Yin Yang*”* theory* and “*Wu Xing*”* theory* [16]. Ancient Chinese physicians believed that a morbid situation appears when the balance of *Yin* and *Yang* or *Wu Xing* in the body was in chaos. Epilepsy is described as *Xia*n or *Xian Zheng* and is caused by interruption of internal body balance induced by pathological factors such as *Feng*, *Huo*, *Tan* and *Yu*, and the rise of *Liver Yang* would occasionally lead to seizures in TCM (Fig. 1) [17]. According to TCM theory, epilepsy primarily affects the heart and brain. In TCM, the pathogenesis of epilepsy is attributed to congenital developmental deficiencies, emotional trauma, dietary irregularities, physical injuries, and other factors. These factors lead to the accumulation of *Feng*, *Huo*, *Tan* and *Yu* (wind, fire, phlegm and stasis), which obscure the heart orifices. Through the meridian transmission system, this disruption further impacts the physiological functions of the five viscera and six internal organs, resulting in the obstruction of clear orifices, *Qi* rebellion, loss of mental clarity, and impairment of vital energy control, ultimately leading to disease onset. Treatment of epilepsy depends very much on its classification within the context of TCM. The earliest known classification of epilepsy was documented in *Zhu Bing Yuan Hou Lun*, attributed to *Cao Yuan Fang* in 610 A.D., delineated five types: “*Yang Dian*”, “*Yin Dian*”, “*Feng* (Wind) *Dian*”, “*Shi* (Wet) *Dian*”, and “*Ma* (Horse) *Dian*” [13]. It is crucial to address this condition using the principles of “*Yin Yang Wu Xing*”. Specifically, excessive *Liver Yang* generates heat, which rises and disturbs the brain and spirit, leading to internal wind. *Liver Yang* rising can result from *Liver Yin* or Blood Deficiency, Phlegm, Blood Stagnation, *Liver Fire*, or *Liver Qi* Stagnation. Epilepsy treatment with TCM follows these fundamental theories, and the interventions including herbal medicine, mineral medicine, animal-derived medicine and acupuncture are used to normalize the interrupted internal balance strictly under the guidance of TCM theory [18].

The recording of ancient TCM prescriptions for epilepsy dates to 200 A.D. in the *Treatise on Fevers*, which is *Chaihu Guizhi Tang* [19]. *Chaihu* (*Bupeuri Radix*) has the function of soothing the liver and regulating *Qi*, and *Guizhi* (*Cinnamomi Ramulus*) acts as warming and *Yang*-dredging which has been reported to treat epilepsy in *the Compendium of Material Medica*. Based on this, a modified prescription known as *Chaihu Longgu Muli Tang* was developed. Besides *Bupeuri Radix* and *Cinnamomi Ramulus*, a total of 11 herbs including *Longgu* (*Keel*), *Muli* (*Ostreae Concha*), *Banxia* (*Pinelliae Rhizoma*), etc. were added to this prescription. Physicians in ancient China believed that this prescription could reconcile *Shaoyang* and calm the nerves. Accumulated literature records as well as research verified that this prescription is somehow effective for epilepsy [20, 21]. Based on kinds of these recordings, some potential medications and treatments have been proposed for treating epilepsy and achieved positive outcomes, which will be discussed in detail in the following sections.

## Recent advances in herbal medicine for treating epilepsy: most common use, also very effective?

**Fig. 2:**
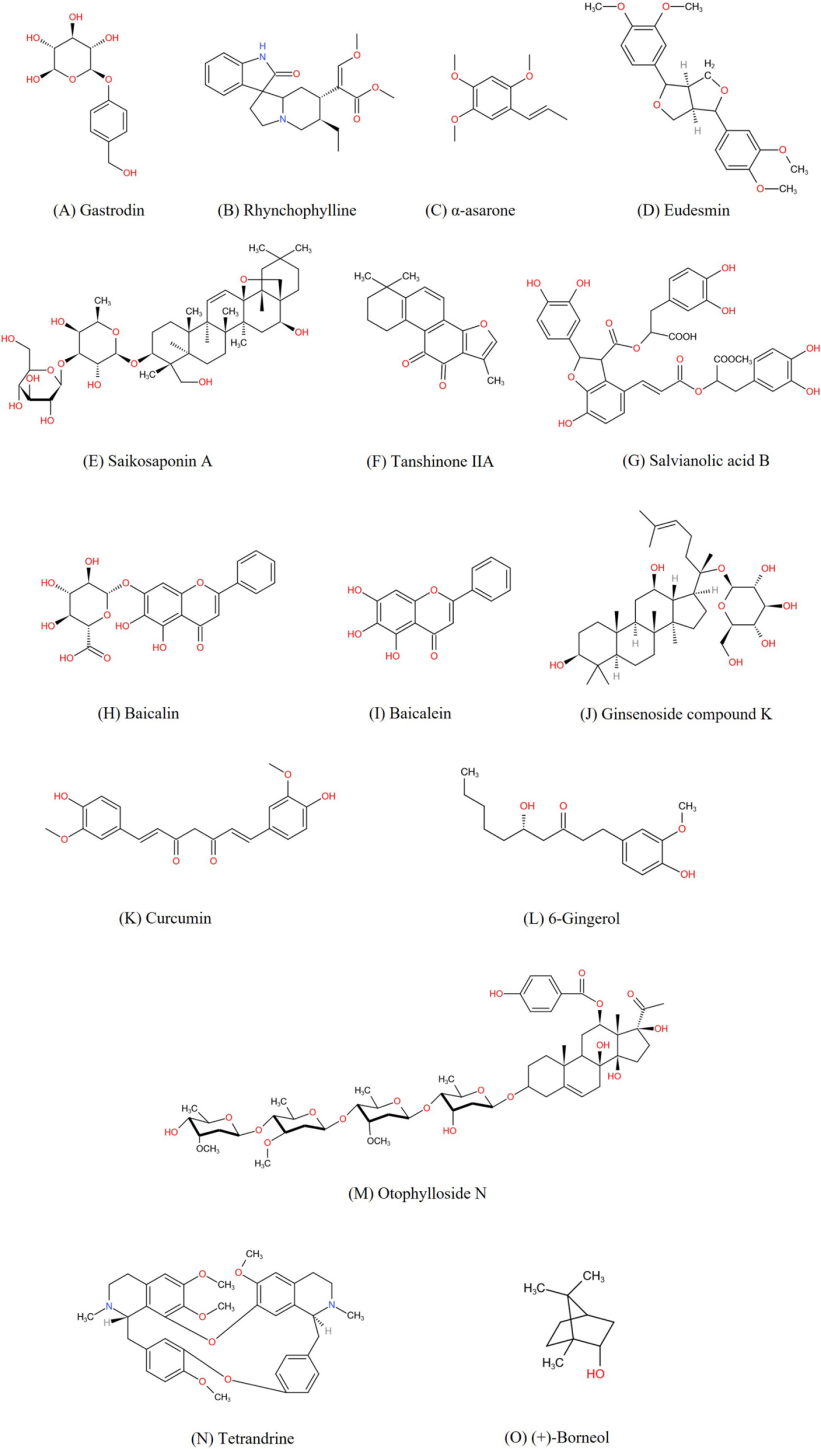
The chemical structures of potential anti-convulsant ingredients derived from TCM. **A** Gastrodin; **B** Rhynchophylline; **C** α-asarone; **D** Eudesmin; **E** Saikosaponin A; **F** Tanshinone IIA; **G** Salvianolic acid; **H** Baicalin; **I** Baicalein; **J** Ginsenoside compound K; **K** Curcumin; **L** 6-Gingerol; **M** Otophylloside N; **N** Tetrandrine; **O** (+)-Borneol

Herbal medicine is the predominant therapeutic option in TCM for epilepsy. Here, eight representative single herbs (Table 1) including their active ingredients (Fig. 2), as well as three herbal prescriptions (Table 2) which have been reported to be potentially anti-epileptic in the last decade are summarized, including their plausible mechanisms of action.

**Table 1:** The summarize of herbal medicine for epilepsy from TCM

Herbs	Chinese Name	Sources	Main active components	Extracting method	Animal models	Possible targets
*Gastrodiae Rhizoma*	Tianma	Dried tuber	Water extract; Gastrodin	Five kilograms of *Gastrodiae Rhizoma* were extracted twice with 35 and 25 L of water by boiling for 1 h and 50 min, respectively	(1) KA model (2) Pilocarpine model (3) PTZ model	(1) JNK signaling pathway (2) Voltage gated sodium channel 1.6 (Nav1.6) (3) Mitogen-activated protein kinases (MAPK) signal pathway (4) GABA_A_ receptor α1 subunit
*Uncariae Ramulus Cum Uncis*	Gouteng	Dry hooked stem and branch	Extract; Rhynchophylline	Eight kilograms of crude *Uncariae Ramulus Cum Uncis* was extracted with 64 kg of 70% alcohol by boiling for 35 min	(1) KA model (2) Pilocarpine model	(1) Toll-like receptors (2) Neurotrophin signaling pathway (3) Nav1.6 (4) NMDA receptor
*Acori Tatarinowii Rhizoma*	Shichangpu	Rhizome	α-asarone; Eudesmin	NA	(1) PTZ model (2) KA model (3) MES model	(1) GABA_A_ receptors (2) Metabolomic level (3) Caspase-3
*Bupeuri Radix*	Chaihu	Dry root	Saikosaponin A	NA	(1) Pilocarpine model (2) PTZ model	(1) NMDA receptor (2) K^+^ channel-interacting proteins and Kv4.2 (3) p-mTOR/p-70S6k pathway (4) Glutamate transportors
*Salviae miltiorrhizae Radix et Rhizoma*	Danshen	Dry root and rhizome	Tanshinone IIA; Salvianolic acid B	NA	(1) PTZ model on zebrafish (2) 6-Hz electrical stimulation model (3) PTZ model (4) Chronic spontaneous model	(1) Caspase-3 (2) Akt/CREB/BDNF signal pathway
*Scutellariae Radix*	Huangqin	Dry root	Baicalin; Baicalein	NA	(1) Pilocarpine-model (2) PTZ model	(1) Oxidative stress mediators (2) TLR4/MyD88/Caspase-3 pathway (3) A1 astrocytes (4) ferroptosis
*Ginseng Radix et Rhizoma*	Renshen	Root and rhizome	Extract; Ginsenoside; Gintonin	Freeze-dried butanol extract	(1) pilocarpine model (2) PTZ model (3) Pilocarpine model (4) KA model	(1) GABA_A_ receptor (2) Inflammatory and oxidant activities
*Curcumae Longae Rhizoma*	Jianghuang	Dry rhizome	Extract; Curcumin	Obtained by subjecting 4 × 100 g of turmeric to hydro-distillation using a Clevenger-type apparatus for 3 h	(1) PTZ model on zebrafish (2) KA model (3) PTZ model (4) FeCl_3_-induced epileptic medel (5) Pilocarpine model (6) Current electroshock seizures (ICES) model	(1) Malondialdehyde (MDA) and nitrite and nitrate (2) Inflammatory cytokines IL-1β and TNF-α (3) Short-term glucose brain hypometabolism (4) Ion channel (CACNA1A and GABRD) protein

**Table 2:** The summarize of TCM prescriptions for epilepsy

Prescriptions	Composition	TCM mechanism	Improved epilepsy-related indicators in animal model
Dian Xian Ning	*Saruma Henryi*, *Pharbitidis Semen*, *Uncariae Ramulus Cum Uncis*, *Acori Tatarinowii Rhizoma*, *Nardostachyos Radix et Rhizoma*, *EuphorbiaeSemen*, *Menthae Haplocalycis Herba, Valerianae Radix et Rhizoma*	Clear phlegm and open orifice, extinguish *Feng* and calm the mind	(1) Inhibit the epileptic behaviors in KA rat model (2) Alleviate the severity in pilocarpine rat model (3) Prolong the latency to seizure in PTZ mice model (4) Reduce the mortality in PTZ mice model (5) Prolong the latency to seizure in PTZ zebrafish model
Ding Xian Pill	*Polygalae Radix*, *Acori Tatarinowii Rhizoma*, *Salviae miltiorrhizae Radix et Rhizoma*, *Pinelliae Rhizoma*, *Citri Reticulatae Pericarpium*, *Fritillariae Cirrhosae Bulbus*, *Poria*, *Ophiopogonis Radix, Arisaema Cum Bile, Bombyx Batryticatus, Scorpio, Gastrodiae Rhizoma, Cinnabaris*, *Succinum*, *Poria Cum Ligno Hospite, Glycyrrhizae Radix et Rhizoma*, *Zingiberis Rhizoma Recens*	Remoe phlegm and extinguish *Feng*, induce resuscitation and calm the mind	(1) Alleviate the seizure severity in PTZ rat model
Xingnaojing Injection	*Moschus*, *Borneol*, *Gardeniae Fructus, Curcumae Radix*	Open the brain orifices, awake the brain, and purge the fire	(1) Shorten the durations of generalized tonic–clonic seizures in the MES mice model (2) Prolong the latency to generalized myo-clonic seizures in the PTZ mice (3) Inhibit the seizure stages, prolong the latency to the first occurrence of both absence and generalized seizures, shorten the seizure durations, decrease the numbers of generalized seizures in the KA mice model

### Single herb—a “Treasure House” for the screen of potential anti-epileptic molecules

#### Gastrodiae Rhizoma

*Gastrodiae Rhizoma* known as *Tianma* in Chinese, is the dried tuber of the orchid *Gastrodia elata Bl*., and is mainly used to dispelling *Feng* and dredging collaterals in TCM theory [22]. In modern medicine, *Gastrodiae Rhizoma* has been reported to have neuroprotective, sedative, anticonvulsant, and antidepressant effects [23–25]. These characteristics make *Gastrodiae Rhizoma* a candidate therapy for diverse neurological diseases including epilepsy [22, 26, 27].

Hsieh et al. performed pharmacological studies on kainic acid (KA)-treated epileptic rats, and observed that oral administration of *Gastrodiae Rhizoma* water extracts at doses of 0.5 g/kg/day and 1.0 g/kg/day for 2 weeks reduced seizure activities, demonstrating its preventive antiepileptic effects [28]. Moreover, Hsieh et al. studies demonstrated that pre-treatment with *Gastrodiae Rhizoma* extracts reversed the reduction of the activity of c-Jun N-terminal kinase (JNK) signal pathway, which was a crucial role in the pathogenesis of glutamate neurotoxicity in the frontal cortex and hippocampus [28, 29]. And compared with the valproic acid (VPA) group (250 mg/kg), the efficacy of high dose *Gastrodiae Rhizoma* extract group was consistent. Post-treatment of *Gastrodiae Rhizoma* extracts also attenuated the elevated JNK signal pathway activity [28].

Gastrodin (GAS) (Fig. 2A), an active ingredient of *Gastrodiae Rhizoma*, has been proved to be anti-epileptic. Shao et al. demonstrated that intracerebroventricular inject 10 mM GAS extended the latency to status epilepticus (SE) and alleviated the electrographic severity of SE caused by pilocarpine [30]. Chen et al. also administered varying doses of GAS in pentylenetetrazole (PTZ) kindled mice, and found that 200 mg/kg GAS decreased seizure intensity and improved the electroencephalogram (EEG) pattern. Both 100 mg/kg and 200 mg/kg prolonged the latency to myoclonic jerk and generalized tonic–clonic seizure [31]. 100 and 200 mg/kg GAS may have therapeutic effect in inhibitory receptor antagonist induced chronic kindling epilepsy model. Yang et al. further demonstrated that a dose of 50 mg/kg GAS effectively reduced seizure severity, decreased the percentage of rats who developed into SE, as well as lowered the number of epileptic spikes in the pilocarpine temporal lobe epilepsy (TLE) rat model [32]. A dose of 50 mg/kg GAS can suppress generalized SE in the acute TLE model induced by the M-type choline receptor agonist [32].

Apart from the anti-epileptic efficacy, the possible mechanism of action for GAS has been proposed in animal studies. Shao et al. observed that GAS exerts an anti-epileptic effect by terminating burst discharges in the medial entorhinal cortex (mEC). Interestingly, the neural inhibitory effect is linked to reduced expression of voltage-gated sodium channel 1.6 (Nav1.6) in glutamatergic neurons following pilocarpine-induced SE [30]. Given that activation of Nav1.6 has been considered to implicated in the generation of epileptic discharges and promotion the occurrence of epileptic seizures [33, 34]. Shao’s findings provided initial evidence for the anti-epileptic effect of GAS being attributed to Nav1.6 inhibition. Besides ion channels, neuroinflammation is also involved in the anti-epileptic effect of GAS. It is verified that GAS can reduce the levels of pro-inflammatory cytokines interleukin-1β (IL-1β) and tumor necrosis factor-α (TNF-α), while increasing the level of anti-inflammatory cytokine interleukin-10 (IL-10) [31]. And GAS could block the mitogen-activated protein kinases (MAPK) signal pathway, and suppress the inflammatory mediators such as cyclic adenosine monophosphate (cAMP)-response element binding protein (CREB) and nuclear factor kappa-B (NF-κB) phosphorylation, in PTZ-treated epileptic mice [35, 36]. This suggests that the anti-epileptic effect of GAS may also be attributed to regulating MAPK-related inflammatory responses. In addition, the inhibitory γ-aminobutyric acid (GABA) receptor might also be involved, as Yang et al. discovered that GAS could reverse the degradation of the GABA_A_ receptor α1 subunit in the pilocarpine-induced TLE model [32, 37].

Compelling evidence from animal models suggests that *Gastrodiae Rhizoma* and its primary active ingredient GAS, traditionally used in TCM for neurological diseases in TCM, exhibit significant therapeutic potential for epilepsy. The anti-epileptic mechanisms of *Gastrodiae Rhizoma* involve the JNK signal pathway, Nav1.6, neuroinflammation and GABA_A_ receptor modulation. However, given the broad impact *Gastrodiae Rhizoma* on neurological functions, careful evaluation of potential side effects is warranted in future translational studies.

#### Uncariae Ramulus Cum Uncis

According to the *Compendium of Materia Medica*, *Uncariae Ramulus Cum Uncis*, derived from dried hooked stems and branches, has been used for centuries in China to suppress hyperactive liver and calm endogenous *Feng*, effectively treating epilepsy.

Since 2011, various pieces of evidence related to the anti-epileptic effect of *Uncariae Ramulus Cum Uncis* has been proposed on experimental models. For instance, Lin et al*.* found that *Uncariae Ramulus Cum Uncis* extracts reduced neuronal death and epileptiform discharges in hippocampal pyramidal neurons in KA TLE model. The relevant mechanisms were also presented, the authors discovered that *Uncariae Ramulus Cum Uncis* extracts decreased the expression of S100B and inhibited glial cell proliferation [38]. Long-term treatment with *Uncariae Ramulus Cum Uncis* extracts reduced the overexpression of S100B and receptor for advanced glycation end products (RAGE), both linked to seizures in KA-treated rats, as further verified by Tang et al. [39]. Their doses of *Uncariae Ramulus Cum Uncis* extracts were 1 g/kg, administered 5 days a week for 2 and 6 weeks, respectively. And the results between *Uncariae Ramulus Cum Uncis* extracts group and VPA group (250 mg/kg) were similar. This suggested that the extracts may effectively treat epilepsy seizures sensitive to ionic glutamate receptors.

Rhynchophylline (Fig. 2B), a derivative ingredient of *Uncariae Ramulus Cum Uncis*, is believed to be the active anti-epileptic ingredient in animal models. Ho et al. reported that rats treated with either oral *Uncariae Ramulus Cum Uncis* extracts (1 g/kg/day) or intraperitoneal rhynchophylline injections (0.25 mg/kg/day) before KA injection showed milder epileptic behaviors compared to the control group [40]. However, the anti-epileptic effects of *Uncariae Ramulus Cum Uncis* extracts and rhynchophylline were overall less effective than VPA treatment (250 mg/kg). And another study confirmed that both pre or post intracerebroventricular injections of rhynchophylline at a concentration of 100 μM reduced the severity of pilocarpine induced-SE in rats [41]. Rhychophylline could treat epilepsy seizures not only be sensitive to ionic glutamate receptors but also choline receptors. These studies not only revealed the anti-epileptic effect of rhynchophylline but also explained its acting mechanism to some extent.

Meanwhile, Ho et al. have proved that *Uncariae Ramulus Cum Uncis* extracts can down-regulate toll-like receptor (TLR) and neurotrophin signaling pathways. Both *Uncariae Ramulus Cum Uncis* extracts and rhynchophylline inhibited KA-induced IL-1β and brain-derived neurotrophic factor (BDNF) gene expression, which were related to enhanced neuronal excitability after KA injection [40, 42, 43]. Shao’s study revealed that the anti-epileptic effect of rhynchophylline may also be related to the functions of Nav1.6 and *N*-methyl-d-aspartic acid (NMDA) receptor. Patch clamp recordings showed that rhynchophylline inhibited the Nav1.6-dependent INaP current and NMDA receptor current. Immunofluorescence analysis confirmed that rhynchophylline decreased the upregulated expression levels of Nav1.6 and NR2B protein, which is a subunit of NMDA receptors [41, 44].

To sum up, *Uncariae Ramulus Cum Uncis* shows great potential as an anti-epileptic agent, with its mechanism of action being linked to S100B, BNDF, Nav1.6 and NMDA receptors. But it should be noted that current studies on *Uncariae Ramulus Cum Uncis* have only focused on KA-treated rodents, and further investigation is needed to evaluate its anti-epileptic effect in other models.

#### Acori Tatarinowii Rhizoma

*Acori Tatarinowii Rhizoma*—the rhizome of *Acorus tatarinowii* Schott, is documented in TCM for its properties to induce resuscitation, eliminate phlegm, resolve dampness and whet the appetite [45]. The first record of *Acori Tatarinowii Rhizoma* was found in *Shen Nong*’*s Herbal Classic* of the Eastern Han Dynasty (nearly 1800 years ago) [46]. *Acori Tatarinowii Rhizoma* is commonly used for resuscitation due to its ability to regulate blood–brain barrier permeability [47]. In TCM practice, it is often combined with *Polygalae Radix*, *Curcume Radix* and other drugs to act as an anti-epileptic role in TCM [48, 49].

The main active ingredient of *Acori Tatarinowii Rhizoma* with anti-epileptic properties is asarone [50]. Huang et al. tested the anti-epileptic effect of alpha(α)-asarone (Fig. 2C) in mice. At 50 mg/kg, α-asarone prolonged seizure latency and reduced mortality in the PTZ model, and decreased seizure susceptibility in the KA model. It has been shown that a therapeutic dose of 50 mg/kg α-asarone is effective in epilepsy models caused by inhibitory receptor agonist and glutamate analogue. Additionally, the study revealed that α-asarone may work by modulating inhibitory GABA_A_ receptors [51–53]. This was confirmed by showing that α-asarone enhanced tonic GABAergic inhibition via extrasynaptic GABA_A_ receptors, suppressing hippocampal pyramidal neuron [51]. Another group further validated that the molecular targets of α-asarone are GABA_A_ receptors and voltage-activated Na^+^ channels [53]. And Zhao et al. further utilized the NMR-Based Metabolomics, demonstrated that α-asarone may normalize metabolic pathways (including alanine, aspartate, and glutamate metabolism; ketone body synthesis and degradation; glutamine and glutamate metabolism; glycine, serine, and threonine metabolism) in PTZ-induced seizure rats, thereby preventing epilepsy [54].

Recently, eudesmin (Fig. 2D), another active ingredient of *Acori Tatarinowii Rhizoma*, has been reported to possess anti-epileptic properties. In 2015, researchers tested the effects of eduesmin in both acute maximum electric shock (MES) and PTZ models. They found that eduesmin (5, 10 and 20 mg/kg) exhibited significant inhibition on seizures induced by either MES stimulation or PTZ injection [55]. The dose range of 5–20 mg/kg not only treated complex seizures induced by electrical stimulation but also partial seizures induced by the GABA receptor antagonist PTZ. But the seizure inhibition rate of each dose of eudesmin group was lower than that of diazepam group (4 mg/kg). And eudesmin could up-regulate the expression of the GABA_A_ receptor and GAD65 (the key synthesis enzyme for GABAergic transmitters), as well as reduce the level of apoptotic caspase-3 [55].

Based on the current evidence, α-asarone and eudesmin are active ingredients of *Acori Tatarinowii Rhizoma*, which exert translational anti-epileptic significance mainly through GABA_A_ receptors. These ingredients are particularly effective for epilepsy types like absence seizures and SE, which benefit from ASDs targeting GABAergic transmissions. Consequently, the future development of new drugs from *Acori Tatarinowii Rhizoma* may also expand to include hypnotic and anti-anxiety purposes.

#### Bupeuri Radix

*Bupeuri Radix*, belongs to *Umbelliferae*, which has been documented in ancient TCM records for its ability to evacuate and relieve fever, as well as soothe the liver and alleviate depression [56]. Previous studies have reported that *Bupeuri Radix* exhibits anti-inflammatory, analgesic, antidepressant, and neuroprotective effects [57–60]. Saikosaponin is the main active ingredient of *Bupeuri Radix* [56].

Recent laboratory studies have confirmed that saikosaponin A (SSA) (Fig. 2E) shows anti-epileptic effects. Yu et al. found that 1 μM SSA inhibited spontaneous recurrent epileptiform discharges (SREDs) and continuous epileptiform high-frequency bursts in an in vitro hippocampal neuron culture (HNC) model of acquired epilepsy (AE) [61]. Furthermore, Hong et al. proved that SSA (1.75, 2.5 and 5 mg/kg, twice a day) decreased the frequency and duration of recurrent seizures as well as alleviated neuronal loss in the li-pilocarpine-treated TLE rats [62]. And only the high-dose group achieved the same effect as the VPA group (100 mg/kg, twice a day). Therefore, the effective dose window of SSA M-type choline receptor agonist-induced TLE rats is 1.75–5 mg/kg. Another study showed that 1.8 mg/kg SSA also reduced seizure severity and duration in PTZ-induced seizures in rats [63].

Electrophysiological evidence showed that SSA inhibited NMDA receptor-mediated current as well as the I_NaP_ mediated by Nav1.6 [61]. Moreover, Hong et al. and Ye et al. demonstrated that SSA also increased the production of inactivating K^+^ currents mediated by K^+^ channel in TLE rats, and suppressed the activation of the p-mTOR/p-70S6k pathway in experimental epilepsy [62, 63]. On another aspect, pretreatment with SSA was found to reduce the glutamate level in the hippocampus of rats by influencing glutamate and aspartate transporter (GLAST) in the PTZ model [64].

Depending on above evidence, it can be deduced that SSA terms as a good anti-epileptic ingredient with various mechanisms. However, it must be acknowledged that current mechanism studies are mainly restricted to in vitro conditions, more direct in vivo evidence is still needed.

#### Salviae miltiorrhizae Radix et Rhizoma

*Salviae miltiorrhizae Radix et Rhizoma* is the dry root and rhizome of *Salvia miltiorrhiza Bge.* of Labiatae, commonly known as *Danshen*. In TCM theory, *Salviae miltiorrhizae Radix et Rhizoma* is used to invigorate blood circulation, remove blood stasis, relieve pain by dredging menstruation, clear heart fire to relieve restlessness, and eliminate carbuncles. Tanshinone IIA (Fig. 2F), a diterpenoid chemical ingredient of *Salviae miltiorrhizae Radix et Rhizoma*, is primarily used to treat cardiovascular and cerebrovascular diseases like atherosclerosis and hypertension due to its anti-inflammatory and endothelial regulatory effects [65–67].

Given that Tanshinone IIA has shown neuroprotective effects in both Alzheimer’s disease and cerebral ischemia, it may also hold promise for treating epilepsy [68, 69]. Tanshinone IIA was firstly reported to exhibit anti-epileptic activity in PTZ induced zebrafish seizure model at the concentrations of 0.1, 1 and 10 mg/kg. It also protected against seizures in the 6-Hz electrostimulation mouse model at doses of 0.5 and 1 mg/kg [70]. Furthermore, in the li-pilocarpine induced chronic spontaneous epilepsy rat model, Tanshinone IIA reduced seizure severities and frequencies in a dose-dependent manner (10, 20, 30 mg/kg) and provided some protection for cognitive function [71]. Meanwhile, compared to the VPA group (189 mg/kg), only the high-dose Tanshinone IIA group significantly reduced seizure severity to a similar extent. Besides, salvianolic acid B (Sal B) (Fig. 2G) is also an active ingredient of *Salviae miltiorrhizae Radix et Rhizoma*. Yu et al. suggested that it significantly reduced seizure scores and inhibited seizure frequency and duration in PTZ-kindled rats when administered orally at 20 mg/kg daily. This effect may be due to inhibiting caspase-3 expression and activating the Akt/CREB/BDNF signaling pathway [72].

Based on recent experimental results, *Salviae miltiorrhizae Radix et Rhizoma* is widely used for preventing and treating cardiovascular and cerebrovascular diseases and has shown anti-epileptic efficacy. Brain trauma, which can lead to epilepsy, often involves significant neuronal apoptosis and loss [73]. It should be noted that the follow-up research on developing candidate anti-epileptic drugs from *Salviae miltiorrhizae Radix et Rhizoma* on preventing trauma-related epileptogenesis.

#### Scutellariae Radix

*Scutellariae Radix* also known as *Huangqin*, is a perennial herb of *Scutellaria* in Labiatae, and its dry roots have been used for clearing heat and toxics in TCM since its first record in *Shen Nong*’*s herbal classi* [74]. Since the twentieth century, *Scutellariae Radix* has been accepted to be neuroprotective effects against neurological diseases such as Alzheimer's disease, Parkinson’s disease etc. [75–79].

Recently, the therapeutic effect of some active ingredients of *Scutellariae Radix* on epilepsy has been successively reported [79–81]. It has been reported that baicalin (Fig. 2H) at 100 mg/kg effectively delays the first limbic seizure, reduces seizure severity, and alleviates SE-induced apoptotic neuronal death in the hippocampus in the pilocarpine model [80]. Recently, Yang et al. further proved that baicalin (50 and 100 mg/kg) has an anti-epileptic effect in PTZ-induced epileptic rats, reducing seizure stages and prolonging seizure latencies. In conclusion, treatment with baicalin at a dose of 100 mg/kg alleviates both SE induced by choline receptor agonist pilocarpine and GABA receptor agonist myoclonic seizures. And in the same study, baicalin treatment also alleviates hippocampal neurodegeneration and improves cognitive impairment in epileptic rats [81].

Possible mechanisms of *Scutellariae Radix* are also presented. Liu et al. found that baicalin reversed oxidative stress mediators like lipid peroxidation, nitrite content, and glutathione levels in the hippocampus, which contribute to neurodegeneration and chronic epilepsy [79]. This indicates that it may play a protective role against pilocarpine-induced oxidative stress [80]. On the other hand, baicalin may also be related to anti-neuroinflammation. Yang et al. discovered that the expression level of TLR4, MYD88 and caspase-3 in the hippocampus were decreased by baicalin. The constructed protein–protein interaction (PPI) network further verified that TLR4, MYD88 and caspase-3 were among the top ten core genes related to baicalin [81]. And a recent investigate showed it may be associated with reducing the polarization of A1 astrocytes. Li et al. discovered that baicalin treatment (50 mg/kg and 100 mg/kg) can improve epileptogenesis and reduce A1 astrocytes in PTZ-induced seizure rats, while also alleviating emotional abnormalities associated with epilepsy [82]. Although the anti-epileptic effect of baicalein was slightly lower than that of VPA group (300 mg/kg), 100 mg/kg baicalein had the same effect as VPA group in relieving mood disorders after epilepsy. Besides, baicalein (Fig. 2I) another bioactive flavonoid, has been reported to have neuroprotective effects in rats with post-traumatic seizures (PTE). Baicalein treatment mitigates seizure behaviors in FeCl_3_-induced PTE mice, by lowering seizure scores, decreasing the occurrence and duration of seizures. Further, the authors discovered that baicalein can inhibited ferroptosis [79].

To sum up, *Scutellariae Radix* is a traditional Chinese herbal medicine for treating neurological diseases. Recent evidence suggests that baicalin and baicalein are the main active ingredients in epilepsy and encephalopathy treatment. The potential mechanisms of action for baicalin may involve antioxidant and anti-neuroinflammation, which can reduce A1 astrocytes. In contrast, baicalein is associated with the suppression of ferroptosis. Based on current evidence, *Scutellariae Radix* likely provides neuroprotection rather than directly inhibiting neuronal excitability. Therefore, drug development from *Scutellariae Radix* may focus on adjuvant or preventive therapies for conditions like acute traumatic injury and infantile febrile convulsion, which can lead to subsequent epileptic seizures [83].

#### Ginseng Radix et Rhizoma

*Ginseng Radix et Rhizoma* (*Araliaceae Juss*) has historically been used in combination with other herbs for various diseases [84]. The medicinal part are the dried root and rhizome of *Panax ginseng* C. A. Mey. Generally, they are used to reinforce vital energy, tonify the spleen and lung, generate fluid and nourish the blood, calm the mind and improve intelligence in TCM. Previous studies have shown that a single dose of ginseng extracts (18 mg/kg) reduced the increase of MRI signal and T2-relaxation time in the hippocampus of rats after SE, and chronic treatment with ginseng extracts also attenuated the early brain damage [85]. Taken that ginsenoside exhibits a neuroprotective effect in neurodegenerative diseases, ginsenoside may have anti-epileptic effect [86]. Zeng et al. found ginsenoside compound K (GCK, a metabolic production of the ginsenoside Rb1, Rb2, and Rc in the intestinal microbiota. Figure 2J) reduced seizure intensities, prolonged the latencies and shortened the seizure durations in rats with PTZ-induced seizures at dosages of 80, 160 and 320 mg/kg. Additionally, GCK delayed the onset of seizures in li-pilocarpine induced SE [87]. And high-dose CCK produced significant antiepileptic effects comparable to those of 400 mg/kg VPA. These shows that GCK at doses of 80–320 mg/kg can alleviate the epilepsy seizures induced by choline agonist pilocarpine and GABA agonist. Especially, the authors discovered that GCK promoted the release of GABA from hippocampal neurons and enhanced the inhibitory synaptic transmission mediated by GABA_A_R, which prompted that GABA receptor may also be a potential target for ginseng [87]. Moreover, gintonin (GT, a novel glycolipoprotein fraction isolated from *Ginseng Radix et Rhizoma*), has also been found to be anti-epileptic in KA-treated mice. Choi JH et al. demonstrated that orally pre-administrated with 50 or 100 mg/kg of GT at 2 h before KA injection dose-dependently lowered the seizure score and alleviated the seizure severity. They confirmed that GT can down-regulate the activation of microglia and astrocyte, lower proinflammatory cytokine/enzyme expression, and boost the nuclear factor erythroid2-related factor 2 (Nrf2)-antioxidant response levels. These findings indicated that GT exerted anti-epileptic properties via anti-inflammatory and antioxidant activities [88].

Although the available evidence is still insufficient, it suggests that ginseng has the potential to treat epilepsy. Currently, ginsenosides are widely used in the clinic, providing convenience for further clinical studies on ginseng’s efficacy against epilepsy. For the laboratory aspect, it is still necessary to carry out in-depth experiments using different models of epilepsy to clarify its pharmacological effects and potential targets.

#### Curcumae Longae Rhizoma

*Curcumae Longae Rhizoma*—the dry rhizome of *Curcuma longa* L. (Zi*ngiberaceae* Martinov), is primarily used in TCM to promote the flow of *Qi* to break blood stasis and dredging meridians to relieve pain. It was firstly recorded in *Wubi Tang* in *Formularies of the Bureau of People’s Welfare Pharmacy*.

Substantial data have shown that *Curcumae Longae Rhizoma* extracts, as well as their ingredients, exhibit anti-epileptic properties. Just as Orellana-Paucar AM et al. proposed that *Curcumae Longae Rhizoma* methanolic extracts at a concentration of 12.5 μg/ml reduced the total seizure activities in zebrafish treated with PTZ [89]. Besides the extracts, curcumin (Fig. 2K) may serve as the active anti-epileptic ingredient of *Curcumae Longae Rhizoma*. As for the TLE model, Kiasalari et al. found that intragastric administration of curcumin (100 mg/kg) alleviated the seizure severity caused by KA injection [90]. Jiang et al. also verified that intraperitoneal injection of curcumin (100 mg/kg) after the cessation of SE inhibited the occurrence of abnormal spikes and spontaneous recurrent seizures [91]. Curcumin is also anti-epileptic in the PTZ model, as proposed by Choudhary KM et al., who found that curcumin (100 and 200 mg/kg) lowered the seizure scores in a dose- and time-dependent manner in PTZ-kindled mice [92]. According to the results, compared with the curcumin group, the phenytoin group (30 mg/kg) had a faster effect on reducing the seizure score. And Kumar et al. found that treatment with curcumin (75 mg/kg) suppressed the epileptiform activities in FeCl_3_-treated rats [93]. Furthermore, Drion CM et al. performed whole-cell recordings and found that curcumin inhibited spontaneous seizure-like events in CA1 neurons [94]. And in the near future, Slowing K et al. detected that oral administration of curcumin (300 mg/kg/day, for 17 days) did not affect the latency to SE onset or the mortality rate in li-pilocarpine induced SE rats. Instead, curcumin can protect body weight and alleviate short-term glucose brain hypometabolism, as well as mitigate signs of neuronal damage and neuroinflammation induced by the SE [95].

Meanwhile, Hashemian et al. prepared curcumin-loaded chitosan-alginate-STPP nanoparticles (NPs) and found that these NPs significantly lowered the seizures stages and shortened the duration of generalized tonic–clonic seizures in PTZ-kindling mice, while also alleviating memory impairment and reducing activated glial cells levels [96]. Bertoncello et al. also found that the micronized curcumin inhibited seizures in the PTZ-induced zebrafish model, but the anti-epileptic effect of curcumin less than VPA (100 mg/kg) [97]. The anti-epileptic effect of liposomal curcumin formulation was verified by Agarwal et al. They found that both oral and intravenous treatment with liposomal curcumin formulation inhibited seizures in the PTZ mice and enhanced the seizure threshold in increasing current electroshock seizures (ICES) mice [98].

The possible mechanisms of curcumin have been partially elucidated; laboratory findings have shown that it influences multiple pathways. Kiasalari et al. found that curcumin attenuated the increase of malondialdehyde (MDA), nitrite and nitrate induced by KA, thereby alleviating the degree of neuronal loss and mossy fiber sprouting (MFS) [90]. Jiang et al. also proved that curcumin reduced the activation of astrocytes and alleviated neuronal loss after SE [91, 96]. Besides, curcumin is able to influence neuroinflammation as discovered by Jiang et al., who found that it inhibited the inflammatory cytokines IL-1β and TNF-α. Consistently, Drion et al. reported that curcumin lowered the expression of inflammatory IL-1β, IL-6 and transforming growth factor β [91, 94]. In addition, Kumar et al. demonstrated that curcumin reduced the expression of α subunit of P/Q type Ca^2+^ channel (CACNA1A) and δ subunit of GABA_A_ receptor (GABRD), which are related to epilepsy in FeCl_3_-induced epileptic rats [93]. And Zhang et al. demonstrated an experimental system that integrated drug affinity responsive target stabilization (DARTS) with affinity chromatography. Their findings revealed USP5, CADPS, and TNR as potential target proteins for curcumin in epileptic mice induced by pilocarpine [99].

To sum, it has been proved that curcumin is anti-epileptic through various administration routes and formulations, suggesting its potential translational significance. But in-depth investigations are still needed.

#### Other herbal medicines

In addition to the TCM herbal medicines mentioned above, some other herbal medicines have also been occasionally reported to have anti-epileptic properties.

Ganoderma lucidum polysaccharide (GLP), an active ingredient of *Ganoderma* was found to inhibit abnormal calcium accumulation and Ca^2+^/calmodulin-dependent protein kinase II α (CaMKIIα) expression in epileptic hippocampal slices induced by low Mg^2+^ [100]. The major active ingredient of *Zingiberis Rhizoma Recens* is 6-Gingerol (6-GIN) (Fig. 2L), which is also reported to be anti-epileptic for PTZ-induced seizures in larval zebrafish [101]. Different doses of 6-GIN significantly decreased PTZ-induced hyper-locomotor activities, and 37.5 µM 6-GIN reduced both the number and mean duration of epileptiform-like discharges. Further mechanism analysis revealed that 6-GIN reduced the Glutamate levels and altered the Glu/GABA ratio [101].

Otophylloside N (OtoN) (Fig. 2M), a pure ingredient isolated from *Cynanchi Otophylli Radix*, has been proved by Sheng et al. to have potent neuroprotective effects. Evidence shows that OtoN (12.5, 25 and 50 μg/ml) reduces the number of high-velocity epileptic seizures in PTZ-treated zebrafish, possibly by attenuating cell injury and decreasing cell apoptosis [102, 103].

Tetrandrine (TTD) (Fig. 2N), an active ingredient isolated from *Stephaniae Tetrandrae Radix*, was discovered by Chen et al. to decrease the frequency of generalized seizures and alleviate the severity of seizure activities in the PTZ rat model at a dose of 30 mg/kg. This effect may be related to inhibiting P-glycoprotein expression in the cortex and hippocampus [104].

Recently, (+)-Borneol (Fig. 2O) enantiomer was shown ameliorating seizures via inhibiting the glutamatergic transmission by our group. We found that 10 mg/kg, 30 mg/kg and 100 mg/kg (+)-borneol can dose-dependently increase the tonic–clonic threshold and reduce the duration of the tonic–clonic seizure in the MES mice model. Meanwhile, in the PTZ mice model, (+)-borneol also prolonged the latency to secondary generalized seizure, and decreased the mortality. However, the anti-epileptic effects of (+)-borneol in MES and PTZ model were not better than that of VPA (200 mg/kg). And in the hippocampal-kindling mice model, the (+)-borneol exhibits protective effects against both kindling-induced epileptogenesis and kindled seizures. (+)-Borneol can also attenuate epileptic seizures in the KA-induced chronic epilepsy mice model, the spontaneous epileptic seizures and generalized seizures can be suppressed. Through a deeper exploration of the mechanism, it is demonstrated that (+)-borneol primarily inhibits spontaneous excitatory postsynaptic currents (sEPSCs) or miniature EPSCs (mEPSCs) [105].

As displayed by recent cumulative laboratory studies, various Chinese herbal medicines and their active ingredients show anti-epileptic effects in both acute and chronic animal models. Their mechanisms of action cover a wide range of targets, including ion channels, synaptic transmission, neuroinflammation, apoptosis, etc. Undoubtedly, the single herbs in TCM provide potential candidate molecules for epilepsy, however, there remain some issues to be addressed: (1) The extracting approaches would influence the abundance of the active ingredients. Exploring the most effective extracting approaches and improving the analysis, identification and separation technologies are sufficient. (2) For those active ingredients, the follow-up studies should be conducted to further test their anti-epileptic efficacy on pharmacoresistant epilepsy models which mimics the most intractable clinical epileptic conditions [106, 107]; on another aspect, due to that related mechanism such as neuroinflammation are reported to be candidate therapeutic target for pharmacoresistant epilepsy [108], those potential anti-inflammatory herb medicines may have a priority for further translational studies. (3) It should be noted that not all the active ingredients have the potentials to be pro-drugs due to their poor bioavailability and pharmacokinetic characteristics, in this case, chemical structure improvement is urgently needed, or whether the active ingredient with anti-inflammatory effect can be encapsulated with targeted materials to explore its anti-epileptic effect.

### TCM prescriptions for epilepsy: various ingredients, effective for different seizure types?

**Fig. 3:**
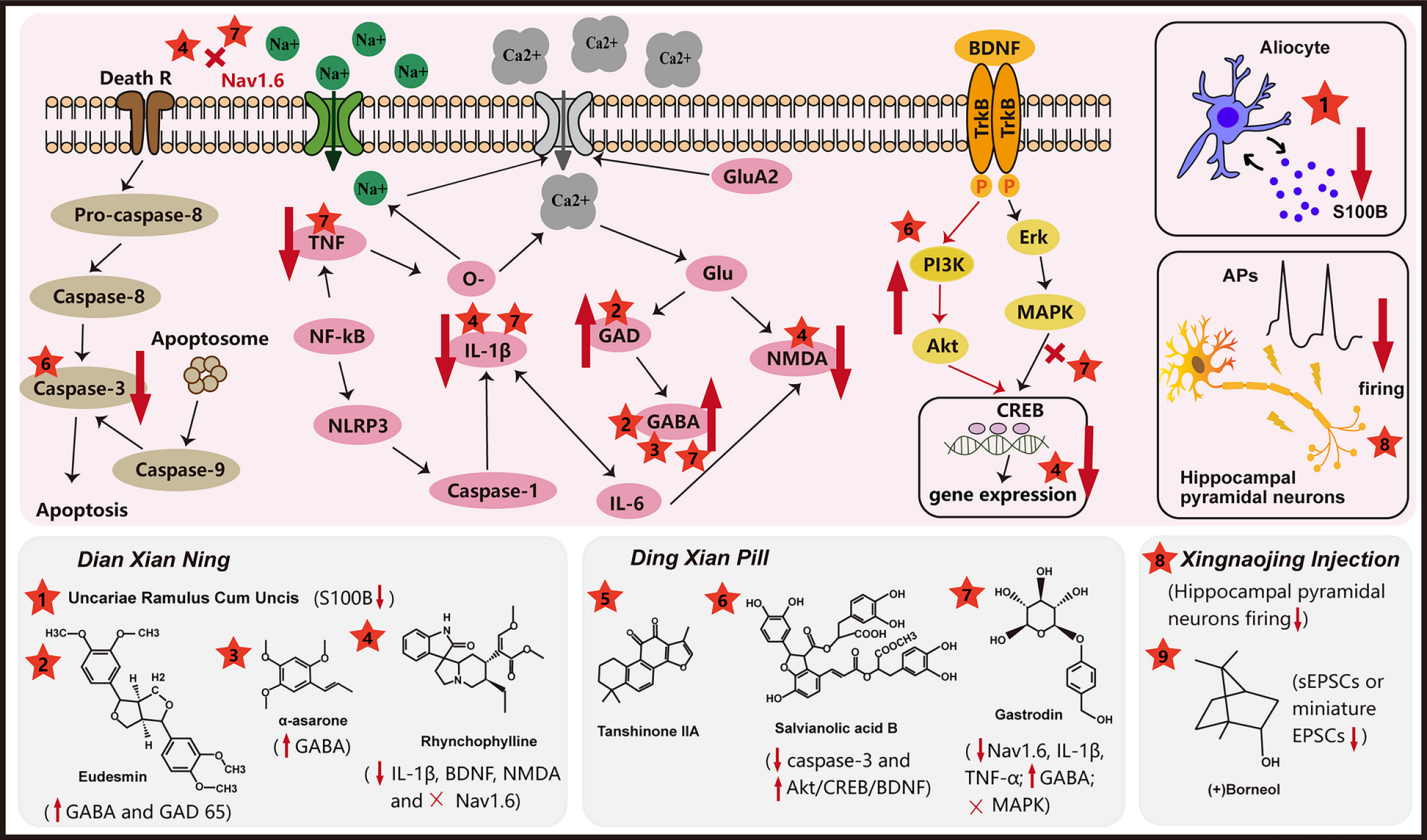
The three representative anti-epileptic TCM prescriptions. First, *Dian Xian Ning* which includes active anti-epileptic ingredients such as Rhynchophylline, eudesmin and α-asarone. *Uncariae Ramulus Cum Uncis* is one of single herb components that can inhibit the overexpression of S100B. Eudesmin can upregulate the expression of GAD and GABA. Rhynchophylline can inhibit IL-1β, BDNF gene expression, downregulate the expression of NMDA and block Nav1.6. Second, *Ding Xian Pill* which contains active ingredients Gastrodin, Tanshinone IIA and Salvianolic acid B. Salvianolic acid B can inhibit the expression of caspase-3 and activate the Akt/CREB/BDNF pathway. Tanshinone IIA is able to inhibit Nav1.6, IL-1β and TNF-α, upregulate the expression of GABA, and block the MAPK signal pathway. Finally, *Xingnaojing Injection* which contains (+)-Borneol can suppress the action potentials of hippocampal pyramidal neurons. (+)-Borneol can modulate the excitatory neural transmissions

It is generally accepted that the pathogenesis of epilepsy is complex, and many epileptogenic factors are involved, which results in various seizure types. Different ASDs correspond to different types of seizures, meaning that one ASD can’t be effective treat all types of seizures. However, unlike current ASDs, TCM prescriptions—a representative medicine in TCM theory composed of multiple ingredients, may act on different targets and have the potential to control different types of seizures (Fig. 3). The following sections focus on the TCM prescriptions that have been proven to be anti-epileptic.

#### Dian Xian Ning prescription

The Dian Xian Ning prescription contains Saruma Henryi, Pharbitidis Semen, Uncariae Ramulus Cum Uncis, Acori Tatarinowii Rhizoma, Nardostachyos Radix et Rhizoma, Euphorbiae Semen, Menthae Haplocalycis Herba and Valerianae Radix et Rhizoma, it is often used for wind-phlegm invading upward in TCM theory [109]. Among those ingredients, Acori Tatarinowii Rhizoma and Uncariae Ramulus Cum Uncis have been proven to be anti-epileptic (see the section on Single herb of this review), and valerian was also reported to contain anti-epileptic active ingredients. In the PTZ-induced zebrafish model, both valerian extracts and valerenic acid were found to prolong the latency to seizures [110]. It has also been shown that valerian extracts can interact with sites of GABA receptors and enhance its inhibitory functions [111, 112]. Moreover, the anti-epileptic mechanisms of Acori Tatarinowii Rhizoma and Uncariae Ramulus Cum Uncis include decreasing the expression of S100B and BNDF, inhibiting the Nav1.6-dependent I_NaP_ current and NMDA receptor current. Taking all these, the Dian Xian Ning prescription shows promise in controlling seizures such as absence epilepsy, status epilepticus, and other seizure types associated with the aforementioned factors.

#### Ding Xian Pill

*Ding Xian Pill*, also named as *expectorant,* is another representative prescription for epilepsy in TCM. This prescription, is known for clearing phlegm, calming the wind, opening the orifices and calming the nerves in TCM theories [113]. It contains *Polygalae Radix*, *Acori Tatarinowii Rhizoma*, *Salviae miltiorrhizae Radix et Rhizoma*, *Pinelliae Rhizoma*, *Citri Reticulatae Pericarpium*, *Fritillariae Cirrhosae Bulbus*, *Poria*, *Ophiopogonis Radix, Arisaema Cum Bile, Bombyx Batryticatus, Scorpio, Gastrodiae Rhizoma, Cinnabaris*, *Succinum*, *Poria Cum Ligno Hospite, Glycyrrhizae Radix et Rhizoma* and *Zingiberis Rhizoma Recens*. Among those ingredients, *Acori Tatarinowii Rhizoma* and *Salviae miltiorrhizae Radix et Rhizoma* have been proved as anti-epileptic (see the section on Single herb of this review).

In PTZ-kindled rats, *Ding Xian Pill* treatment could alleviate the seizure severities, inhibit the hippocampal abnormal discharges and reverse cognitive impairment caused by seizures [114]. According to the authors, this effect might be related to Egr3-GABRA4, NRG1-ErbB4, ERK-Arc and COX2-P-gp signal pathways [114]. On the other hand, researchers revealed that the *Ding Xian Pill* was able to increase the expression of glutamate transporter to decrease the glutamate level [115]. These suggested that *Ding Xian Pill* was the potential to block the propagation of seizures originating from the focus.

#### Xingnaojing Injection

*Xingnaojing Injection* (XNJ) which is derived from the classic TCM formula “*An Gong Niu Huang Wan*”. It consists of *Moschus*, *Borneol*, *Gardeniae Fructus* and *Curcumae Radix* is renowned for the efficacy in acute ischemic injury. According to the TCM theory, XNJ can open the brain orifices, awake the brain, and purge the fire. This laid a theoretical basis for XNJ to treat epilepsy. Our group have recently demonstrated the anti-epileptic effects of XNJ in several acute mice models, including MES, PTZ, and KA models, but the overall effect was not as good as VPA (200 mg/kg) [116]. In the MES mice model, 10 ml/kg and 20 ml/kg XNJ could shorted the durations of generalized tonic–clonic seizures; in the PTZ mice model, 20 ml/kg XNJ could significantly prolonged the latency to generalized myo-clonic seizures; also, 20 ml/kg XNJ could inhibited the seizure stages, delayed the occurrence of the first seizures or generalized seizures, shorted the seizure durations, decreased the numbers of generalized seizures in the acute KA model. Furthermore, we employed in *vitro* electrophysiological recordings, proved that XNJ directly inhibited both the spontaneous and evoked action potentials of hippocampal pyramidal neurons, thus suppress the seizures.

The forementioned findings demonstrate a centralized representation of XNJ and (+)-Borneol, which is one component of XNJ, both show the potential therapeutic efficacy in various animal models [105]. In comparison to XNJ, (+)-Borneol exhibited a broader spectrum of activity towards chronic epilepsy models, thereby highlighting the multi-target advantage of TCM prescriptions in disease treatment.

Although identifying the real active components from TCM prescriptions is still technically difficult, just as the concept of polypharmacy in the clinical use of ASDs, it is highly likely that a TCM prescription that contains various anti-epileptic ingredients may have advantages to be useful for different seizure types. Therefore, we propose that subsequent investigations need further optimization in developing new anti-epileptic prescriptions, aiming to act on different mechanisms and reduce possible side effects.

## Acupuncture for epilepsy: acting as a kind of neuromodulation?

**Fig. 4:**
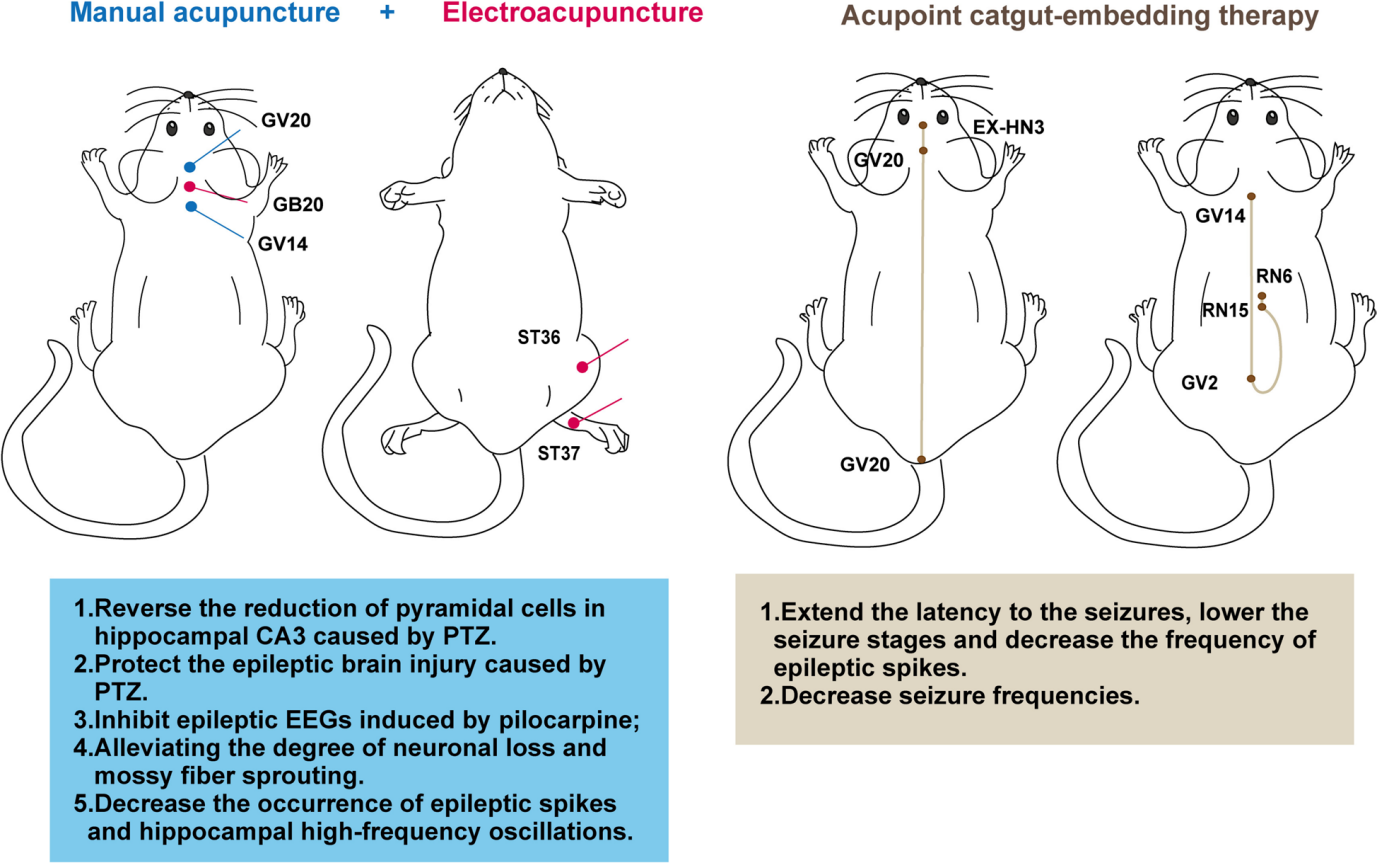
The therapeutic effect of acupuncture on epilepsy. Acupuncture therapy including manual acupuncture stimulation, electroacupuncture and acupoint catgut-embedding. Typical acupoints can produce therapeutic effects on epilepsy

Besides pharmacotherapies, acupuncture is also an effective intervention for neurological disease in TCM. As one of the representative intangible cultural heritage, acupuncture can relieve symptoms by stimulating specific acupoints [117]. Here, we mainly summarize the findings regarding acupuncture therapy at different acupoints for epilepsy (Fig. 4).

### Manual acupuncture

In TCM, the theories of acupuncture for treating epilepsy include coordinating the viscera, smoothing *Qi* and blood, harmonizing *Yin Yang*, and acupuncture exhibits the advantages such as minimal adverse reactions and long-term availability. Recently, the role of acupuncture in epilepsy has been related to its neuroprotection effect. Yang et al. found that manual acupuncture at the “*Baihui*” (GV20) and “*Dazhui*” (GV14) acupoints reversed the reduction of pyramidal cells in the hippocampal CA3 caused by PTZ-induced seizures in rats. However, combining acupuncture with phosphatidylinositol-3-kinase (PI3K)/protein kinase B (Akt) signaling antagonist showed no protective effect, suggesting that acupuncture’s protective effect may involve the intracellular apoptotic PI3K/Akt pathway [118, 119]. Furthermore, Yang et al. have confirmed that acupuncture at “*Baihui*” (GV20) and “*Dazhui*” (GV14) could protect against PTZ-induced epileptic brain injury in rats by bidirectionally regulating the expression of anti-apoptotic glucose regulatory protein 78 (Grp 78) and apoptotic C/EBP homologous protein (CHOP) [120, 121]. *Lai Xinsheng*, a famous acupuncture expert in China, pointed out that acupuncture can be used in epileptic attacks and intermittent periods, and also emphasized the significance of combination therapies of acupuncture and ASDs in treating epilepsy [122]. According to a meta-analysis of the effectiveness of acupuncture for epilepsy, the overall clinical efficacy of acupuncture or its combination with ASDs is superior to conventional medication, highlighting the advantages of acupuncture for treating epilepsy [123].

### Electroacupuncture

Electroacupuncture (EA), based on traditional acupuncture, uses parameterized electrical stimulation instead of manual operations. Similar to neuromodulation, EA treatment shows potential in inhibiting seizures. Yi et al. found that low-frequency (10 Hz) electroacupuncture at *Fengchi* acupoint (GB20) can inhibit epileptic EEGs induced by pilocarpine in rats [124]. Liao et al. discovered that long-term electrical stimulation (6 weeks) of the auricular and EA at *Zusanli* and *Shangjuxu* acupoints (ST36-ST37) in KA-treated rats, reduced pathological MFS levels, epileptic spikes and hippocampal high-frequency oscillations [125]. Another study by Lin et al. confirmed that 2 Hz EA at *Zusanli* and *Shangjuxu* acupoints (ST36-ST37) effectively reduced the discharge in the hippocampal CA1 region in KA-induced epileptic rats [126].

The EA treatment for epilepsy may be anti-neuroinflammatory. Liao et al. discovered that EA decreased the level of COX-2 in the CA1 region, as well as reduced the number of reactive astrocytes and S100B levels, indicating that EA at *Zusanli* and *Shangjuxu* (ST36–ST37) acupoints exhibited anti-neuroinflammatory effect [125]. Another study by Lin et al. showed that the effects of EA on KA-induced epileptic rats at auricular and *Zusanli* or *Shangjuxu* (ST36–ST37) might be attributed to the influence of the transient receptor potential ankyrin 1 (TRPA1), as well as other factors such as PKCε, PKCα and pERK1/2 [126].

Unlike drugs that can directly target specific molecules, the core mechanisms of acupuncture appear similar to current neuromodulation therapies (such as vagus nerve stimulation). It exerts therapeutic effects by stimulating specific acupoints according to meridian theories. Although the specific structural basis of meridians remains unclear, evidence supports the distant effects of acupuncture through somatosensory autonomic nerve reflexes [127]. Since the 1970s, studies have found that this kind of reflex has somatic regional specificity, but the neuroanatomical basis underly this somatic regional specificity is still unclear [128, 129]. A recent study found that low-intensity (0.5 mA) acupuncture stimulation at *Zusanli* acupoint (ST36) is sufficient to activate the “vagus nerve adrenal NPY^+^ medullary cells” pathway, while the high-intensity (3 mA) acupuncture stimulation at *Zusanli* (ST36) can activate the “spinal cord sympathetic” reflex [130, 131]. These encouraging results certify the existence of neural circuits between peripheral acupoints and intracerebral inferior colliculi, paving a new way for analyzing the underlying neural circuitry basis of anti-epileptic effect induced by EA stimulation.

### Acupoint catgut-embedding therapy

Acupoint catgut-embedding therapy is another treatment similar to acupuncture in TCM for neurological diseases including epilepsy [132]. The procedure of acupoint catgut embedding involves the insertion of medical catgut, an allogeneic protein capable of inducing an immune response, into specific acupoints to provide continuous stimulation [132]. In an animal experiment, Jin et al*.* tested the anti-convulsant effect of acupoint catgut-embedding on a rat model induced by penicillin, the authors performed the catgut implantation at “*Yintang*”, “*Baihui*” and “*Changqiang*” acupoints (EX-HN3, GV20, GV1) [133]. Behavioral and EEG recordings showed that pretreatment with acupoint catgut-embedding could extend the latency to the seizures, lower the seizure stages and decrease the frequency of epileptic spikes. Encouragingly, according to their study, the anti-epileptic effect of acupoint catgut embedding was similar to that of sodium valproate [133]. This anti-epileptic effect is further verified in the clinical studies, Mao et al*.* divided 52 patients with generalized seizures into acupoint catgut-embedding combined ASDs group and ASDs monotherapy group [134]. Acupoint catgut-embedding was performed at *Dazhui*, *Yaoshu*, *Jiuwei* and *Qihai* acupoints (GV14, GV2, RN15, RN6). By recording the seizure occurrences as well as the epilepsy score (the patients state of consciousness, disturbance of consciousness, duration of forced convulsion and EEG). It was found that there was a significant difference in epilepsy scores between the two groups, but not in seizure frequencies, indicating that acupoint catgut-embedding relieves the seizure symptoms and promotes the quality of life for PWEs [134].

It is indeed true that acupuncture has emerged as an effective treatment for epilepsy. Laboratory evidence obtained through advanced techniques, confirms that acupuncture stimulates peripheral-central neural circuits, indicating it may have similar effects and mechanism to neuromodulation methods used for pharmacoresistant epilepsy. Delighted by the advantages of acupuncture which are non-invasive and relatively safe, future efforts should be paid to uncovering the neural circuitry basis of evidence-based anti-epileptic acupoints, so that to guide clinicians to select more effective parameters for acupuncture.

## TCM on epileptic comorbidities: we care about more

Besides controlling seizures, another practical dilemma in clinical epilepsy is the management of epileptic comorbidities. The concept of comorbidity, referring to any other concurrent disease which are usually concealed by diagnosed diseases, was first proposed in the 1970s [135, 136]. The comorbidities in epilepsy include depression, anxiety, dementia, stroke, migraine, and heart disease et al. Among these, the prevalence of gastrointestinal and respiratory diseases is eight times higher in epilepsy than that in the general population [137]. Epileptic comorbidities severely affect the quality of life for PWEs. Current ASDs are usually helpless in this situation, in addition, continuous medication may even aggravate the comorbidities. Searching effective interventions for both epilepsy and comorbidities remains essential.

**Fig. 5:**
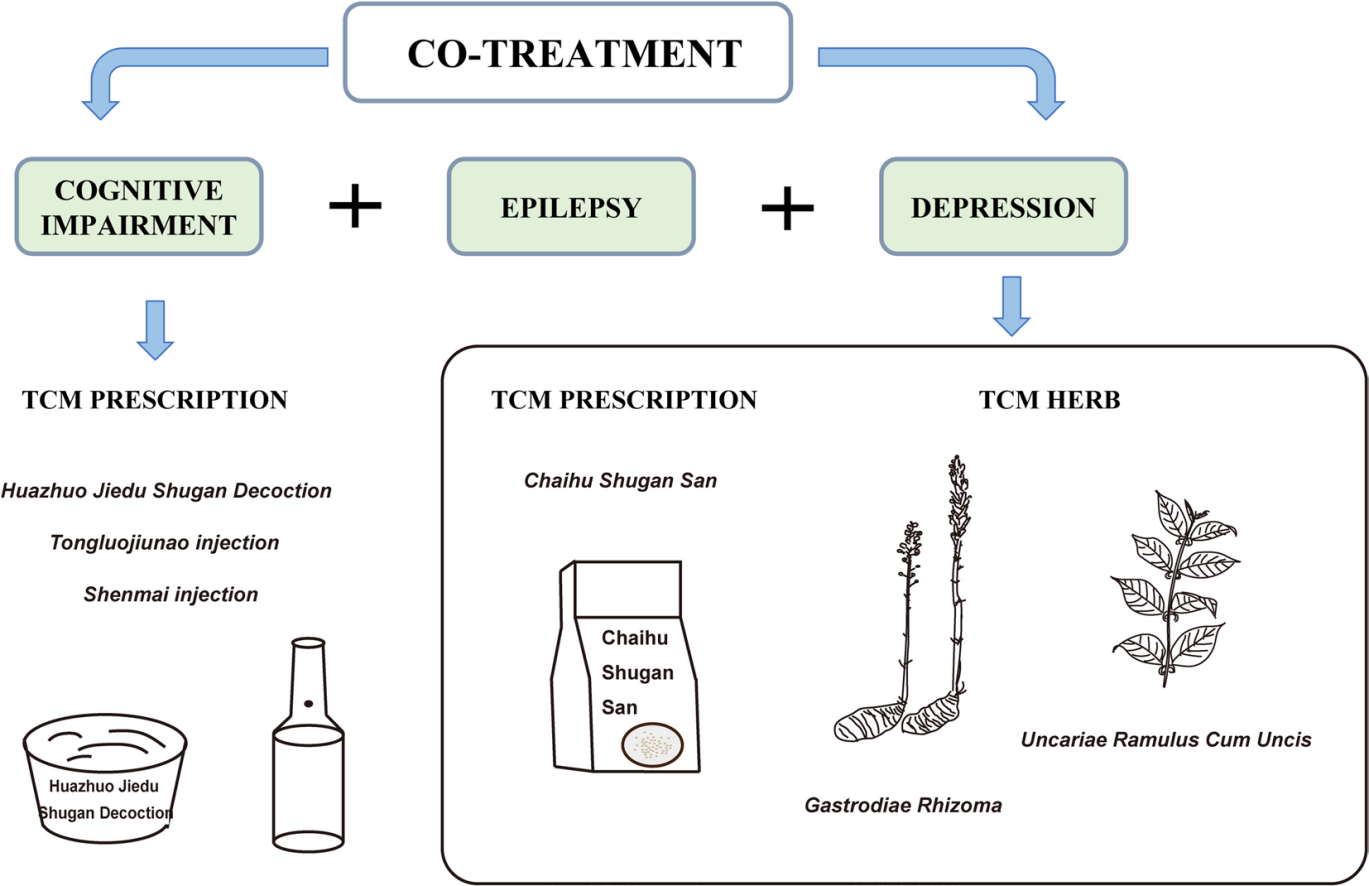
Effective TCM interventions for epileptic comorbidities. Some representative TCM prescriptions or herbal medicines is potentially effective for both control epileptic symptoms and comorbidities. TCM prescriptions for the treatment of epilepsy comorbid with cognitive impairment are *Huazhuo Jiedu Shugan Decoction*, *Tongluojiunao Injection* and *Shenmai Injection*. TCM prescription for the treatment of epilepsy comorbid with depression is *Chaihu Shugan San*. TCM herbs for the treatment of epilepsy comorbid with depression are *Gastrodiae Rhizoma* and *Uncariae Ramulus Cum Uncis*

In TCM theories, different diseases may share same pathological factors, this cognition raised up a concept which is the “*homotherapy for heteropathy to different diseases*”. Numerous ancient literatures have proved that anti-epileptic medicines can also be beneficial for other neurological diseases. These lead researchers to consider that certain interventions derived from TCM can be effective for both epilepsy and its concurrent comorbidities. Encouragingly, recent evidence proposes that TCM may offer an alternative way to managing some epileptic comorbidities (Fig. 5). Taken that TCM herbs and prescriptions have treatment characteristics of various ingredients and multi-target, suggesting that TCM therapy has certain advantages in the treatment of epilepsy comorbidity.

### Depression

Depression commonly cooccurrence with epilepsy, and the prevalence of depression in pharmacoresistant epilepsy is approximately 54% [138, 139]. Current clinical antidepressants, mainly selective serotonin reuptake inhibitors, may increase seizure risk in a dose-dependent manner [140, 141]. TCM prescriptions with the advantages of acting on multi-targets may overcome this dilemma. *Chaihu Shugan San* has been proven to be effective and safe in the treatment of depression [142]. Early studies have shown that the co-occurrence of epilepsy and depression is linked to the activation of the activation of the 5-hydroxytryptamine 1A (5-HT1A) receptor [143]. Research evidences have showed that *Chaihu Shugan San* can increase the expression of 5-HT1A receptor, and thus promoting cell proliferation in dentate gyrus of hippocampus in epileptic rats with a depressive state, induced by pilocarpine and chronic mild stress (CMS), effectively improving some depressive symptoms [144].

There are also some single compounds derived from TCM which are both anti-epileptic and antidepressant. Anti-epileptic GAS which is the active ingredient in *Gastrodiae Rhizoma* is also reported to have antidepressant effects via protecting hippocampal neural stem cells from proinflammatory cytokine IL-1β [145]. Besides, Xian et al. also found that isorhynchophylline, a hydroxyindole alkaloid derived from *Uncariae Ramulus Cum Uncis* which is anti-epileptic, showed desirable antidepressant effect in mice experiencing chronic unpredictable mild stress (CUMS) through influencing neurotrophic factors and PI3K/Akt/GSK-3bp pathway [146]. Regarding that a certain number of medicines in TCM has been shown to be anti-depressive [147]. Searching useful medications from TCM which are both anti-epileptic and anti-depressive seems highly possible.

### Cognitive impairment

Cognitive impairment in epilepsy is not rare in both younger and elder PWEs. In children with epilepsy, repetitive seizures usually result in intellectual disability and special learning disabilities [148]. Elder patients are also in danger with an increased risk of dementia [149]. *Huazhuo Jiedu Shugan Decoction* (HJSD), composed of *Scutellariae Radix*, *Acori Tatarinowii Rhizoma*, *Bupleuri Radix*, *Gynostemma Pentaphyllum* and *Basil* is proposed to be effective on seizure-induced cognitive impairment. Experimental results showed that HJSD could significantly improve the performances of cognitive tasks in epileptic animals. Further molecular evidence revealed that HJSD increased the number of immune response cells of adenylate cyclase (AC), cAMP and CREB, this may be due to the regulation of AC-cAMP-CREB signaling pathway in hippocampal neurons [150].

Another report showed that *Tongluojiunao* (TLJN), an injection composed of Chinese herbal *Notoginseng Radix et Rhizoma* and *Gardeniae Fructus*, is useful in reducing the levels of excitatory neurotransmitters related to neuronal death, and improving cognitive impairment in Alzheimer’s rats [151]. For those pharmacoresistant epilepsy patients who have undergone epileptic surgery, postoperative cognitive dysfunction (POCD) is one of the main complications. Studies have shown that preoperative treatment of *Shenmai* injection and postoperative treatment of *Shenfu* injection are beneficial for the recovery of consciousness and prevent the occurrence of POCD in elderly rats [152]. Indicating that these medications are potentially useful for postoperative cognitive impairment in PWEs.

To sum up, TCM shows its unique advantages in the management of epileptic comorbidities (mainly depression and cognitive impairment). Therefore, in the future, one concept can be considered in the treatment of epilepsy along with other comorbidities by TCM, that is selecting specific components known to be effective for various symptoms to developing new prescriptions.

## Future prospects and conclusions: what need to do next?

At present, great advances have been achieved in the last one decade in searching TCM-based anti-epileptic interventions including single herbs, prescriptions, acupuncture, and other therapies, and the potential acting targets involve voltage-gated ion channels, neurotransmissions, neuroinflammation pathways, apoptotic factors et al. However, despite the existing shreds of evidence, there remain five issues that need to be carefully addressed: (1) the precise mechanisms of TCM interventions are still not completely illustrated; (2) the appropriate epileptic symptoms for different TCM interventions are remained to be determined; (3) whether TCM can retard epileptogenesis rather than just inhibiting seizures remains doubtable; (4) the absence of these comparisons between TCM and modern ASDs hinders the ability to emphasize the distinct advantages and competitiveness of TCM in clinical setting, which highlighted the importance of using ASDs as positive control to objectively evaluate the efficacy of TCM therapies; (5) the potential interactions between TCM herbs and conventional ASDs have not been addressed. Given that many epilepsy patients are on long-term antiseizure medications (ASDs), understanding these possible interactions is crucial to prevent adverse effects or diminished efficacy.

From the perspective of TCM, treatment must achieve “treating both the symptoms and the root causes”, correspondingly, we propose further exploration of potential interventions targeting pathogeneses involved in epileptogenesis from TCM, but not just focus on neural inhibitory mechanisms. The second prospection is to perform more in-depth studies on current effective medications and interventions to not only test their efficacy on multiple pharmacoresistant epilepsy models, but also reveal their possible targets. An analysis of the limitations inherent, such as discrepancies between animal models and clinical practice, as well as the representativeness and adequacy of the sample size. Given that the ingredients of TCM pharmacotherapies are complex, clarifying their acting mechanisms is a necessary stepping stone to transfer TCM from evidence-based to mechanism-based. And robust clinical evidence is essential to substantiate the real-world efficacy and safety of TCM interventions. The lack of prominence given to such trials undermines the persuasiveness of translating TCM treatments from bench to bedside. Last but not the least, the majority of current evidences are obtained from animal models. Although many studies utilize animal models, they do not thoroughly examine the distinctions between these models and clinical reality. Such distinctions include variations among animal species and the differences between experimental disease induction methods, clinical etiologies. Thus, more standard randomized control trials (RCTs) are needed to push forward the clinical translation of TCM interventions for epilepsy.

In conclusion, this review provides a comprehensive overview of the advancements in TCM interventions for epilepsy, followed by the hypothesis of their possible mechanisms. At last, crucial issues such as future pharmacology studies, translational significance and in-depth investigations into underlying mechanisms are proposed to guide future endeavors aimed at developing novel TCM-based treatments for epilepsy.

## Data Availability

No data was used for the research described in the article.

## Abbreviations

ASDs: Anti-seizure drugs
TCM: Traditional Chinese Medicine
PWE: Patients with epilepsy
KA: Kainic acid
JNK: C-Jun N-terminal kinase
GAS: Gastrodin
VPA: Valproic acid
SE: Status epilepticus
PTZ: Pentylenetetrazole
EEG: Electroencephalogram
TLE: Temporal lobe epilepsy
mEC: Medial entorhinal cortex
Nav 1.6: Voltage-gated sodium channel 1.6
IL-1β: Interleukin-1β
TNF-α: Tumor necrosis factor-α
IL-10: Interleukin-10
MAPK: Mitogen-activated protein kinases
cAMP: Cyclic adenosine monophosphate
CREB: CAMP-response element binding protein
NF-κB: Nuclear factor kappa-B
GABA: γ-Aminobutyric acid
RAGE: Receptor for advanced glycation end products
TLR: Toll-like receptor
BDNF: Brain-derived neurotrophic factor
NMDA: *N*-Methyl-d-aspartic acid
MES: Maximum electric shock
SSA: Saikosaponin A
SREDs: Spontaneous recurrent epileptiform discharges
HNC: Hippocampal neuron culture
AE: Acquired epilepsy
GLAST: Glutamate and aspartate transporter
Sal B: Salvianolic acid B
PPI: Protein–protein interaction
PTE: Post-traumatic seizures
GCK: Ginsenoside compound K
GT: Gintonin
Nrf2: Nuclear factor erythroid2-related factor 2
NPs: Nanoparticles
ICES: Increasing current electroshock seizures
MDA: Malondialdehyde
MFS: Mossy fiber sprouting
CACNA1A: α Subunit of P/Q type Ca2+ channel
GABRD: δ Subunit of GABAA receptor
DARTS: Drug affinity responsive target stabilization
GLP: Ganoderma lucidum polysaccharide
CaMKIIα: Ca2+/calmodulin-dependent protein kinase II α
6-GIN: 6-Gingerol
OtoN: Otophylloside N
TTD: Tetrandrine
sEPSCs: Spontaneous excitatory postsynaptic currents
mEPSCs: Miniature EPSCs
XNJ: Xingnaojing Injection
PI3K: Phosphatidylinositol-3-kinase
Grp 78: Glucose regulatory protein 78
EA: Electro-acupuncture
TRPA1: Transient receptor potential ankyrin 1
5-HT1A: 5-Hydroxytryptamine 1A
CMS: Chronic mild stress
CUMS: Chronic unpredictable mild stress
HJSD: Huazhuo Jiedu Shugan Decoction
AC: Adenylate cyclase
TLJN: Tongluojiunao
POCD: Postoperative cognitive dysfunction
RCTs: Randomized control trials
